# Evidence for an emotional adaptive function of dreams: a cross-cultural study

**DOI:** 10.1038/s41598-023-43319-z

**Published:** 2023-10-02

**Authors:** David R. Samson, Alice Clerget, Noor Abbas, Jeffrey Senese, Mallika S. Sarma, Sheina Lew-Levy, Ibrahim A. Mabulla, Audax Z. P. Mabulla, Valchy Miegakanda, Francesca Borghese, Pauline Henckaerts, Sophie Schwartz, Virginie Sterpenich, Lee T. Gettler, Adam Boyette, Alyssa N. Crittenden, Lampros Perogamvros

**Affiliations:** 1 Sleep and Human Evolution Lab, University of Toronto, Mississauga, Canada; 2 Department of Anthropology, University of Toronto, 3359 Mississauga Road, Mississauga, ON L5L 1C6 Canada; 3 Department of Basic Neurosciences, University of Geneva, Geneva, Switzerland; 4 School of Medicine, Johns Hopkins University, Baltimore, MD USA; 5 Department of Human Behavior, Ecology and Culture, Max Planck Institute for Evolutionary Anthropology, Leipzig, Germany; 6 Department of Archaeology and Heritage, Institute of Resource Assessment, University of Dar es Salaam, Dar es Salaam, Tanzania; 7 Laboratoire National de Santé Publique, Brazzaville, Republic of the Congo; 8 Department of Anthropology, University of Notre Dame, Notre Dame, IN USA; 9 Department of Anthropology, University of Nevada, Las Vegas, USA; 10 Department of Psychiatry, Center for Sleep Medicine, University Hospitals of Geneva, Geneva, Switzerland; 11 Department of Psychiatry, Center for Sleep Medicine, 2 Chemin du Petit-Bel-Air, 1226 Thônex, Switzerland

**Keywords:** Psychology, Anthropology

## Abstract

The function of dreams is a longstanding scientific research question. Simulation theories of dream function, which are based on the premise that dreams represent evolutionary past selective pressures and fitness improvement through modified states of consciousness, have yet to be tested in cross-cultural populations that include small-scale forager societies. Here, we analyze dream content with cross-cultural comparisons between the BaYaka (Rep. of Congo) and Hadza (Tanzania) foraging groups and Global North populations, to test the hypothesis that dreams in forager groups serve a more effective emotion regulation function due to their strong social norms and high interpersonal support. Using a linear mixed effects model we analyzed 896 dreams from 234 individuals across these populations, recorded using dream diaries. Dream texts were processed into four psychosocial constructs using the Linguistic Inquiry and Word Count (LIWC-22) dictionary. The BaYaka displayed greater community-oriented dream content. Both the BaYaka and Hadza exhibited heightened threat dream content, while, at the same time, the Hadza demonstrated low negative emotions in their dreams. The Global North Nightmare Disorder group had increased negative emotion content, and the Canadian student sample during the COVID-19 pandemic displayed the highest anxiety dream content. In conclusion, this study supports the notion that dreams in non-clinical populations can effectively regulate emotions by linking potential threats with non-fearful contexts, reducing anxiety and negative emotions through emotional release or catharsis. Overall, this work contributes to our understanding of the evolutionary significance of this altered state of consciousness.

## Introduction

Why do humans dream? As a product of the brain’s neurophysiology, our species can produce hallucinatory experiences during sleep. These dream experiences represent an altered state of consciousness. Why is it that we exhibit this altered state of consciousness rather than experiencing sleep in total perception quiescence? Research investigating dream content reveals that the dream state of consciousness, which is most often expressed in rapid-eye movement (REM), appears to be preoccupied with world simulation with content often reflecting the self’s social realities^1,2^, social networks^3,4^, and dangers^5^. Yet, whether dreams could enhance cognitive, affective, or social adaptation has been a question of active debate for decades.

A common framework for explaining the function of dreams is provided by *simulation theories*, which are based on the premise that dreams have a biological function and reflect selective pressures and fitness enhancement in the evolutionary past via altered states of consciousness^6^. Accordingly, dreams are credible real-world analogs^7–14^ that prime the individual for corresponding contexts encountered in waking life. From this perspective, it has been argued that the phenotypic expression of dreaming could meet the necessary criteria for evolution by natural selection^15^.

### Dream simulation and emotion regulation

Emerging work integrating neuroscience and dream content analysis suggests that emotional experiences are a crucial part of the virtual-world simulation of dreams and support an adaptive process that contributes to the resolution of emotional distress and preparation for future affective reactions^6,16–19^. In this context, the threat simulation theory^6^ and social simulation theory^9^ posit that dreams are biased to simulate threatening and social situations respectively. Such a mechanism would, in turn, promote adjusted behavioral responses in real-life situations^5,9^. Other studies have also supported the idea that past negative memories are reprocessed and combined in dreams with new, realistic, and safe contexts, suggesting the possibility of desensitization^20,21^ or extinction^17^ functions for dreaming. Functional dreams could thus expose us to threatening situations while providing us with efficient solutions to these situations. Such a process may facilitate the resolution of current social and emotional internal conflict^16,22^, a process also called emotional *catharsis*^23^, and the reduction of next-day negative mood^24^.

Together, these proposals and empirical observations suggest a potential core function of dreams via simulating distress in a safe environment to help process threats in beneficial ways; as such, functional dreams would strongly contribute to efficient *emotion regulation* in wakefulness^18^. These mechanisms seem to be impaired in clinical populations, such as patients with nightmare disorder^17,25^ and anxiety disorders^26^—two pathologies characterized by less efficient *fear extinction*^17,27^.

Indeed, anxiety is considered a maladaptive emotional response implicating dysfunction of inhibitory (extinction) learning^27^, and the persistence of the fear response across time. We would thus expect that dreams with high levels of anxiety and negative emotions in the presence of a threat, as those found in clinical populations, would not serve the emotional processing function of dreams, as no emotional resolution is achieved. Critically, Revonsuo posited that the adaptive emotional function of dreams may be particularly relevant to contemporary small-scale societies facing routine ecological risks such as infectious disease and predation, as the emotional simulating mechanism would be fully activated in the face of the kinds of challenges within their environment^6^. Although there is some preliminary evidence for this argument^5,28, 29^, such arguments have yet to be comparatively tested with large, multicultural datasets.

### The importance of cross-cultural testing of dream content

The major challenge to the scientific investigation of dream function remains a sampling problem. To date, most dream studies have been conducted in the Global North—and primarily in the U.S. and European settings with samples of limited socio-economic and racial/ethnic breadth. Thus, one critical challenge to overcome limitations in past dream-based research, is to test the function of dreams by generating dream content variation among diverse populations’ socio-ecological experiences. This may be due in part to the interest of sleep researchers in pairing such work with sleep-based physiological techniques (i.e. polysomnography) that have been historically limited to lab settings (but see^30^ for field-based methods in human biology and sleep research that are gaining momentum). While historically dreams have been the subject of anthropological investigation^28,31–33^, this ethnographic work is largely descriptive. Hence, much of the dream data are generated from studies that represent a very narrow range of human experiences for select populations (e.g., college undergraduates) at specific historical moments (e.g., between 1970 and 1990) in particular locations (e.g., U.S., Europe) and under similar societal and economic contexts (e.g., educated, high income).

There is a dearth of direct empirical tests of the evolutionary function of dreams, including comparative perspectives that would enable us to assess variation across cultural and ecological contexts in relation to dream content^9^. For example, smaller-scale societies that engage in mixed-subsistence foraging (i.e., hunt and gather for a large part of their diet), often differ from other smaller-scale societies in important ways. The depth and breadth of egalitarianism (i.e., cultural values and practices aimed at the treatment of all individuals as equal, often with norms around avoidance of prestige and hierarchy) in many sub-tropical foraging populations is intertwined with norms of cooperative pooling of time and energetic resources, such as to help provision and care for children^34–39^. Such forms of egalitarianism and extensive cooperation in resource sharing and family life are thought to be critical to survival and reproduction.

In contemporary populations, including the Hadza of Tanzania and BaYaka of the Republic of the Congo forager communities we focus on here, these cooperative subsistence and social dynamics necessarily place a strong emphasis on the importance of face-to-face supportive relationships for day-to-day health, well-being, and even survival^35,38–49^. These communities share some broad socioecological similarities in terms of (i) continuous environmental exposure to key stimuli—such as ambient light and temperature cues—known to drive circadian entrainment (e.g., circadian driven fluctuations have been shown to influence central characteristics of dream reports^50^), (ii) gender divisions of foraging and household labor (though varying in their intensity between the BaYaka and Hadza), (iii) ecological risk in the form of predation exposure by way of large animals, pathogens and parasites, and (iv) norms regarding egalitarianism and generous resource sharing behaviors^39–44,51,52^.

The community-oriented interpersonal behaviors characteristic of BaYaka and Hadza and their maintenance require high degrees of emotion regulation and social problem solving. Unlike the experience of many individuals in populations from the Global North, these foragers’ daily interactions are repeated with the same network of cooperative partners throughout their lives. Additionally, although precise estimates are difficult to assess, mortality rates are relatively higher in subsistence-level societies compared to populations with better access to emergency care and biomedical treatment^41^—a factor that may be relevant in evaluating the possible threat simulation function of dreams. Thus, foragers may experience greater threat *and* community oriented responses to threat in their dreams. If an adaptive function of dreams is to reinforce or rehearse such day-to-day, prosocial (i.e., community-oriented) interactions, particularly with trusted kin, then people in BaYaka and Hadza communities will have a higher representation of those interactions and family members in their dreams than would typical populations in the Global North who reside in more individualistic societies.

### Hypotheses and predictions

Here, we compare the dreams of two foraging communities—the BaYaka and Hadza—to non-clinical and clinical (i.e., with nightmares and social anxiety) populations from the Global North. First, because of their strong egalitarian social norms and high levels of daily face-to-face interpersonal support from trusted family and friends, we predict that the dream content of both forager groups will have a greater frequency of community-oriented behaviors when compared to dreamers in the Global North. Second, given that both forager groups experience greater early-to-midlife mortality—subsequently leading to a greater chance of an individual losing their own life, the life of offspring, kin, or friends—we predict a greater frequency of threat related dream content relevant to mortality. Third, we expect that foragers’ dreams will serve an efficient emotion regulation function, where threats are associated with new, non-fearful contexts/efficient solutions^17^, and, thus, with lower anxiety/negative emotions in dreams. Finally, we predict that the Nightmare group will have greater levels of negative emotions in dreams and that the student group, associated with COVID-19 pandemic, as well as the social anxiety group, will be characterized by greater anxiety in dream content. By comparing these groups, we can better understand the role of culture and environment in shaping the human experience of dreaming.

## Material and methods

### Participants

In all, individuals from two sub-Saharan foraging egalitarian communities with low degrees of market integration, the Hadza and BaYaka and from three high income capitalistic populations (including non-clinical and clinical populations) totaling 234 participants contributed 896 dreams (see Table 1 for summary details).

**Table 1:** Sample summary description of dream participants.

Community	Country	Participants	Mean age	Dream reports
Non-clinical control	Switzerland, Belgium	103	22.1	356
Social Anxiety Disorder	Switzerland	37	24.4	184
Nightmare Disorder	Switzerland	33	26.3	113
University of Toronto Students	Canada	24	21.9	168
BaYaka	Democratic Republic of Congo	19	42.3	27
Hadza	Tanzania	18	42.7	48

#### Global North data collection and characteristics

Data from the Global North populations were drawn from previously published studies done in Switzerland, Belgium, and Canada. The Switzerland and Belgium samples were generated between 2014 and 2022^25,26,53–55^ and included data from three groups: a non-patient group of young healthy participants, patients suffering from social anxiety disorder (SAD), and patients suffering from nightmare disorder. Participants in these studies all kept the same sleep and dream diary (for details see^18^). During the night or every morning, upon spontaneous awakening, the participants were asked to report whether they had a dream with or without recall or no dream at all. They also reported the presence of specific emotions thanks to dichotomous questions (presence/absence); in total, eleven emotions could be reported. A twelfth choice was reserved to the “absence of emotions”. In the last section of the dream diary, they were also asked to freely write down the dreams they had experienced during their sleep.

The non-clinical reference control group in the Global North includes 219 participants (123 females). A subset of 103 participants, aged between 16 and 40 years old (M = 22.1, SD = 7.9), had dream word counts equal to or greater than 20 words and were included in the dream analysis (word average per dream = 78.2, SD = 66.0). All participants followed a constant sleep schedule during the days preceding the experiment to assess the mean sleep duration and exclude any circadian disturbance or sleep disorder. People suffering from mental disorders were excluded. Ethical approval was granted by the committee of the Faculty of Medicine of the University of Liege and by the Ethical Committee of the Canton of Geneva.

Dreams were also collected from patients suffering from social anxiety disorder (SAD) according to *The Diagnostic and Statistical Manual of Mental Disorders* (DSM5)^26,56^. SAD is characterized by a persistent amount of fear when confronted with social situations^57^. Forty-eight subjects (32 females) were included in the final sample, after assessment of their social anxiety disorder level. The dream diary was filled every morning upon awakening for 2 weeks. Three hundred twenty-four (324) dream reports were collected (6.75 dreams per participant). A subset of 37 participants, aged between 16 and 40 years old (M = 24.4, SD =7.9), had dream word counts equal to or greater than 20 words and were included in the dream analysis (word average per dream = 76.9, SD = 56.7). Ethical approval was granted by the Ethical Committee of the Canton of Geneva, Switzerland (“Commission Cantonale d’Ethique de la Recherche sur l’être humain”).

Additionally, dreams were collected in individuals suffering from nightmare disorder^25^. In total, 36 patients (27 females) were included. All of them suffered from nightmare disorder according to DSM5 with at least moderate severity (> 1 episode per week). Every morning upon awakening participants filled in a dream diary for 2 weeks. One hundred thirty-four (134) dream reports were collected (3.72 dreams per participant). A subset of 33 participants, aged from 20 to 35 years old (M = 26.3, SD = 8.4), had dream word counts equal to or greater than 20 words and were included in the dream analysis (word average per dream = 43.5, SD = 23.8). Ethical approval was granted by the Ethical Committee of the Canton of Geneva, Switzerland (“Commission Cantonale d’Ethique de la Recherche sur l’être humain”).

Altogether, the Belgian and Swiss studies had 924 dream reports collected from the dream diary over 397 nights (4.2 dreams per participant on average). Of those dreams the number that were included in the final analysis with words counts equal to or above 20 are as follows: *control N* = 356, *Nightmare Disorder N* = 113, and *SAD* = 184.

Students at the University of Toronto contributed dream reports (*N* = 184) collected during the fourth wave of the COVID-19 pandemic, where the proliferation of COVID-19 variants was of major concern in Ontario, Canada, as announced by the Public Health Agency of Canada (Statistics Canada, 2021). In total, 24 students (21 females) aged from 19 to 25 years old (M = 21.9, SD = 5.5) were included. Ethics was approved and attained by the University of Toronto REB (RIS Human Protocol Number 39768). During this time, self-rated mental health was below national average (< 50%), and 82% of the Canadian population that were eligible for vaccination were fully vaccinated, however restrictions were still imposed in most areas, including mask-wearing, and limiting contacts. Thus, explorations of evolutionary theories on dream functions may have special relevance during the COVID-19 pandemic^58,59^. The final number of dreams equal to or above 20 words and included in the analysis was *N* = 168 (word average per dream = 120.6, SD = 44.4).

#### Global South data collection and characteristics

Data were collected over different time periods by different experimenters. Hadza participants (*N* = 18) were surveyed by DRS in January and February of 2016 and BaYaka participants (*N* = 19) by AHB, SLL, VM, and MSS in June and July 2017. Hadza participants were aged between 18 and 68 years old (M = 42.7, SD = 8.5) and BaYaka participants were aged between 27 and 70 years old (M = 42.3, SD = 10). Combined, we collected a total of 101 dream reports (2.16 dreams per participant and a word average per dream = 38.7, SD = 18.9). The Hadza contributed 48 dream reports (female dreams = 12, male dreams = 36; word average per dream = 44.4, SD = 20.6); all Hadza dreams were equal to or greater than 20 words and were included in the analysis. The BaYaka (*N* = 19) contributed 53 dream reports (females dreams = 26, male dreams = 27); twenty-seven BaYaka dreams were equal to or greater than 20 words (word average per dream = 28.7, SD = 9.1) and were included in the analysis.

Dream reports were collected in the field using a modified Most Recent Dream (MRD) method^60^ as a template for questionnaires, and in practice (as the indigenous populations could not write) were a daily verbally administered dream diary. The instructions, given by field researchers in morning after a sleep period, requested the participant to recall whether they dreamt the previous night. If subjects answered in the affirmative, they were then asked to recount the details of the dream using the MRD method template. The report was expected to be detailed, including a description of the dream's setting, the people involved (their age, sex, and relationship to the participant), and any animals present in the dream. Participants were also instructed to describe their emotions during the dream and whether it was a positive or negative experience. This method is ideal for use in small-scale societies because it is a fast, inexpensive, and reliable way to obtain large samples of dream reports. For both forager groups, dream content was translated by the aid of a multilingual field assistant at the time the dream was recorded. Importantly, it is essential to note that, as both the MRD modified and verbally administered dream diary (Global South) and the classic Dream Diary method (Global North) recorded dreams of the previous night, they shared a similar approach and were directly comparable. Additionally, both were administered shortly after awakening from sleep on the same day as the dream, thereby minimizing potential memory biases^61^.

For work with the Hadza, IRB approval was granted from the University of Nevada, Las Vegas (2014) and verbal consent for participation was asked to each participant in Swahili, the second language of the Hadza community. All research was performed with approval of the government of Tanzania, via the Tanzanian Commission for Science and Technology (COSTECH) and the Tanzanian National Institute for Medical Research (NIMR). For the BaYaka, village council consent for this study was obtained at a community meeting in 2015. Subsequently, community consent was annually renewed. Verbal consent was provided by each participant following recruitment into this study. Approval to conduct research in the Republic of the Congo was given by The Centre de Recherche et D’Edudes en Sciences Sociales et Humaines. Ethics approval was obtained from Duke University (2017), the University of Notre Dame (2017), and the University of Cambridge (2017).

All methods were performed in accordance with the relevant guidelines and regulations, and informed consent was obtained from all participants.

### Dream text analysis

LIWC-22 is an acronym for Linguistic Inquiry and Word Count, and it is a text analysis software program that can return results for up to 90 different variables or categories^62^. The English text analysis strategy employed the LIWC-22 Dictionary. This internal dictionary is comprised of over 12,000 words, phrases, and emoticons, which have been carefully selected and categorized into sub-dictionaries to assess various psychosocial constructs. Essentially, the LIWC-22 software program is designed to map linguistic constructions to important psychosocial theories and constructs, and thus, target words contained in texts that are read and analyzed by LIWC-22 are used for this purpose.

In this study, the dream texts were translated and transcribed into English, and preprocessed into four super-categories—*Community-oriented* (by grouping the LIWC categories: social, family, moral, friend, and prosocial)*, Threat* (by grouping the LIWC categories: conflict and death)*, Negative emotions* (encompassing the category: negative emotions), and *Anxiety* (encompassing the category: anxiety). To create an outcome variable for statistical models (see *section ‘Modelling'*), we summed the number of words of each category in each dream text. Examples of the *Community-oriented* target words were: care, help, thank, please, parent, mother, father, baby, honor, deserve, judge, you, we, he, she. Examples of the *Threat* target words were: fight, killed, attack, death, dead, die, kill. Examples of the *Negative emotions* target words were: bad, hate, hurt, tired. Examples of the *Anxiety* target words were: worry, fear, afraid, nervous. The LIWC-22 Dictionary provides a systematic and reliable approach to text analysis^63^ and has been widely used in other word-based dream content analyses^25,64,65^.

### Modelling

To assess the predictors of the four response variable categories (*Community-oriented, Threat, Negative emotions, Anxiety* dream content) by population (BaYaka, Hadza, Nightmare, SAD, Students, and Control) we used a linear mixed effects model, built using the lme4 package and model averaged using the MuMin package^66^. To normalize the count data for each category, we square root transformed the response variable^67,68^. Finally, we made statistical inferences using a combination of standardized coefficients, confidence intervals, and p-values. We controlled for the fixed effects of age, number of dream reports, word count and sex as well as subject ID (to control for repeated measures) as a random effect. After assessing information criterion, models including the number of dream reports and age as fixed effects differed little from models without them, and so we removed them from final analysis. To increase the power of the model to identify the predicted patterns in the data, we obtained coefficients based on optimization of the log-likelihood using shrinkage, which incorporates measurement error into the regression model and improves less certain estimates by pooling information from more certain estimates^69^.

The non-patient sample from the Global North was used as a model reference category (i.e., a group that is used as a point of comparison for other groups in a statistical analysis) so effect-size estimates for each population are predicted differences in counts of dream content compared to this sample.

The dream content models were fit as follows:

$${\varvec{M}}{\varvec{o}}{\varvec{d}}{\varvec{e}}{\varvec{l}}:\boldsymbol{ }dream content \sim Population+Sex +Word Count+ \left(1|Subject\right)$$

$$\begin{aligned} {\text{Response}}_{i} & \sim N\left( {\alpha_{j[i]} + \beta_{1} ({\text{WC}}) + \beta_{2} ({\text{Sex}}_{{{\text{female}}}} ),\sigma^{2} } \right) \\ \alpha_{j} & \sim N\left( {\gamma_{0}^{\alpha } + \gamma_{1}^{\alpha } ({\text{Population}}_{{{\text{BaYaka}}}} ) + \gamma_{2}^{\alpha } ({\text{Population}}_{{{\text{Hadza}}}} ) + \gamma_{3}^{\alpha } ({\text{Population}}_{{{\text{Nightmare}}}} ) + \gamma_{4}^{\alpha } ({\text{Population}}_{{{\text{SAD}}}} ) + \gamma_{5}^{\alpha } ({\text{Population}}_{{{\text{Students}}}} ),\sigma_{\alpha j}^{2} } \right),\;{\text{for}}\;{\text{ID}}\,{\text{j}} = 1, \ldots {\text{J}} \\ \end{aligned}$$

The full dataset, along with all meta-data and more detail of each variable, is available in the Open Science Framework (OSF) data repository: https://osf.io/7n6kf/.

## Results

### Community-oriented’ dream content is greatest in BaYaka

**Figure 1:**
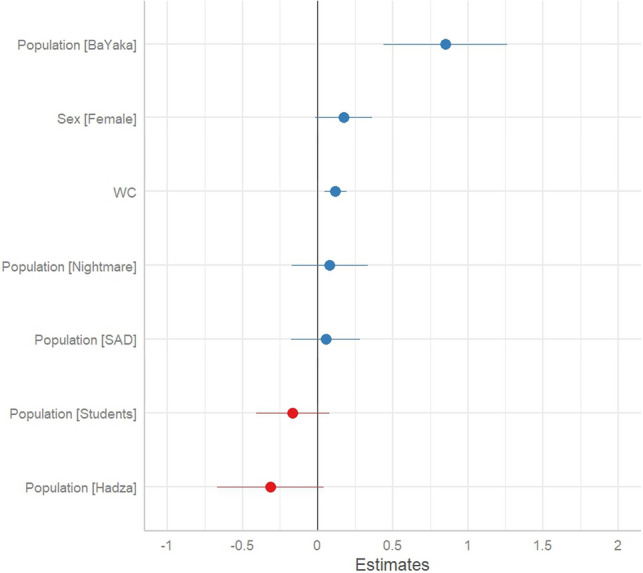
Prosocial dream estimates plot.

Amongst all sampled populations, the BaYaka showed greater community-oriented dream content than all group samples from Global North populations and Hadza population, after adjusting for sex, word count, and subject ID. As shown in Table 2, and displayed in Fig. 1, after factor correction, the BaYaka sample positively drives community-oriented dream content. Additionally, women’s dream reports and word count were drivers of the response variable (Table 2). As ethnographic data, we present a few such examples here:

*‘I was walking in the forest with my two adult daughters and found a porcupine in a trap and brought it back to the village to eat it. It was a good dream’*

*‘I was net hunting with my family (including extended family camp) and we caught many animals so he had to make a smoker "bota" to smoke all of them’*

**Table 2:** The effect of predictor variables on *prosocial dream content*.

Predictor	Estimate (S.E.)	Confidence interval	P value
Population: BaYaka	0.14 (0.03)	(0.072, 0.214)	< 0.01*
Population: Hadza	− 0.08 (0.04)	(− 0.160, 0.004)	0.06
Population: Nightmare	0.03 (0.04)	(− 0.058, 0.111)	0.54
Population: SAD	0.02 (0.05)	(− 0.071, 0.115)	0.65
Population: Students	− 0.06 (0.05)	(− 0.158, 0.033)	0.20
Sex: Female	− 0.060 (0.03)	(− 0.006, 0.157)	0.07
Word Count	− 0.042 (0.04)	(0.048, 0.193)	0.01*

The *Control* is the reference category for *Population*, and *Male* is the reference category for *Sex*. Positive coefficients indicate greater prosocial dream content, while negative coefficients indicate lesser prosocial dream content. The Asterix signifies a significant p value.

### ‘Threat’ dream content is greatest in BaYaka and Hadza

**Figure 2:**
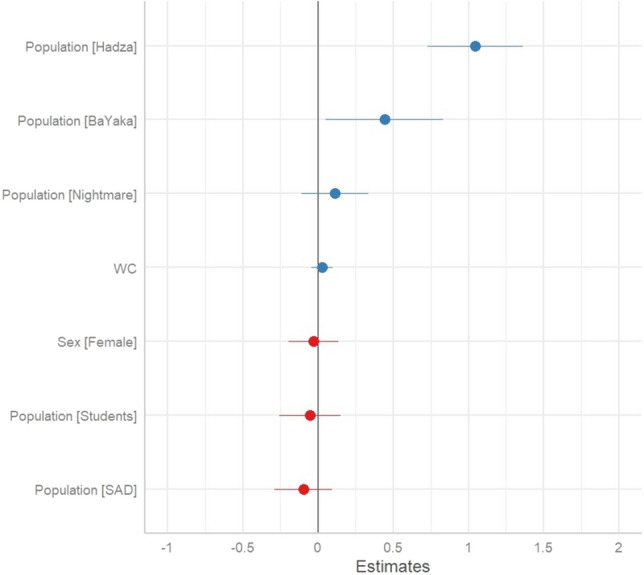
Threat dream estimates plot.

After adjusting for sex, word count, and controlling for repeated measures of the subject ID, both the BaYaka and Hadza samples had higher levels of threat dream content compared to the Global North groups. This is shown in Table 3 and depicted in Fig. 2. Thus, belonging to the BaYaka or Hadza community is associated with a greater probability of experiencing threatening dream content. No other factors were found to significantly influence threat dream content.

**Table 3:** The effect of predictor variables on *threat dream content*.

Predictor	Estimate (S.E.)	Confidence interval	P value
Population: BaYaka	0.07 (0.03)	(0.007, 0.140)	0.02*
Population: Hadza	0.24 (0.04)	(0.166, 0.306)	< 0.01*
Population: Nightmare	0.03 (0.04)	(− 0.040, 0.106)	0.37
Population: SAD	− 0.04 (0.04)	(− 0.117, 0.038)	0.32
Population: Students	− 0.02 (0.04)	(− 0.094, 0.061)	0.64
Sex: Female	− 0.01 (0.04)	(− 0.083, 0.059)	0.74
Word Count	− 0.02 (0.04)	(− 0.046, 0.095)	0.49

The *Control* is the reference category for *Population*, and *Male* is the reference category for *Sex*. Positive coefficients indicate greater threat dream content, while negative coefficients indicate lesser threat dream content. The Asterix signifies a significant p value.

Importantly, several dream reports gathered among the Hadza community demonstrated high threat situation to which a positive, emotionally cathartic resolution was found. For example:

*‘I dreamt I was being chased by a herd of elephants; I was in Nyanza, which is open flat savanna land. I ran and found a small cave which was too small for the elephants to follow. I escaped’.*

*‘I was chased by an elephant in the bush around camp. I was with four unfamiliar women. I escaped by running into the mountains’.*

*‘I dreamt I was in the forest and the military was chasing me with guns and he climbed a tree to get away.’*

*‘I was chased by a leopard in nearby mountains. I began by hunting but realized that I was the hunted. I was alone but I escaped’.*

Moreover, in some Hadza dream reports, a solution to a threat was found through social support:

*'I dreamt I fell into a well that is near the Hukumako area by the Dtoga people. I was with two others and one of my friends helped me get out of the well.'*

*‘I dreamt a buffalo hit me. I was in Numbeya bushland where we look for honey. I was looking for the "small honey". There was another man called January and he came and helped me’*

*‘I dreamt a Toga not from this camp (who) took a knife and a person he didn't know from another camp. After I told the guy to stop, he left our Sengele camp.’*

### 'Negative emotion’ dream content is greatest in Nightmare disorder sample

**Figure 3:**
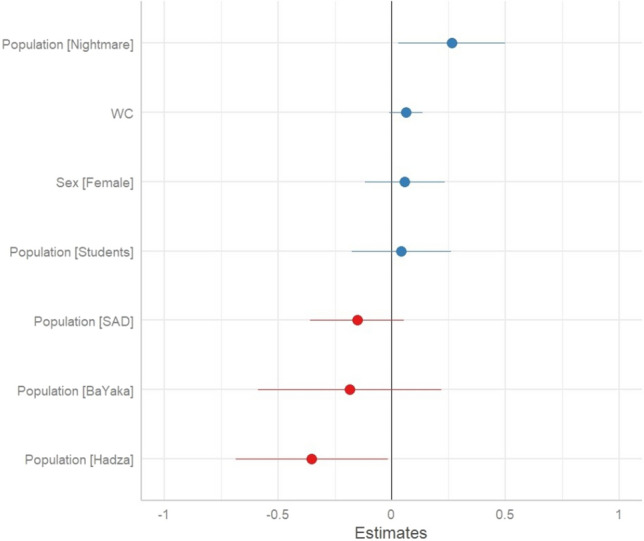
Negative emotions dream estimates plot.

After adjusting for sex, word count, and subject ID, the sample of patients from the Global North in the *Nightmare Disorder* group had higher levels of dream content with negative emotions compared to the reference group (Table 4 and Fig. 3). Conversely, the Hadza exhibited significantly fewer negative emotion words in their dream content than the reference group. No other groups differed from the reference group, as shown in Table 4 and depicted in Fig. 3. The following dream reports demonstrate high fear without resolution in the Nightmare Disorder group:

*‘My mom would call me on my phone and ask me to put it on speakerphone so my sister and cousin could hear. Crying she announced to us that my little brother was dead. I was screaming in sadness and crying in pain.’*

*‘I was with my boyfriend, our relationship was perfect and I felt completely fulfilled. Then he decided to abandon me, which awoke in me a deep feeling of despair and anguish.’*

*‘I remember in my dream is that I was sitting at a table, in one of the secret rooms, across from a middle-aged man who said he was my uncle (he did not look like any of my uncles), and he was over 100 years old but looked like he was in his 50s. He looked like evil characters from movies. He said he was going to kill me after he went to speak with other people in the other room to admit his secret and then come to kill me. After he left the room, I got up and saw that the door was not fully closed. My thought was that I had to go fight him and then I woke up before I could approach the door.*

**Table 4:** The effect of predictor variables on *negative emotions dream content*.

Predictor	Estimate (S.E.)	Confidence interval	P value
Population: BaYaka	− 0.04 (0.04)	(− 0.106, 0.032)	0.30
Population: Hadza	− 0.08 (0.04)	(− 0.160, − 0.012)	0.02*
Population: Nightmare	0.08 (0.04)	(0.005, 0.162)	0.04*
Population: SAD	− 0.06 (0.04)	(− 0.146, 0.022)	0.15
Population: Students	0.02 (0.04)	(− 0.061, 0.110)	0.57
Sex: Female	0.03 (0.04)	(− 0.049, 0.107)	0.46
Word Count	0.06 (0.04)	(− 0.008, 0.137)	0.08

The *Control* is the reference category for *Population*, and *Male* is the reference category for *Sex*. Positive coefficients indicate greater negative emotional dream content, while negative coefficients indicate lesser negative emotional dream content. The Asterix signifies a significant p value.

### ‘Anxiety’ dream content is greatest in the Canadian (COVID-19 pandemic era) student sample

**Figure 4:**
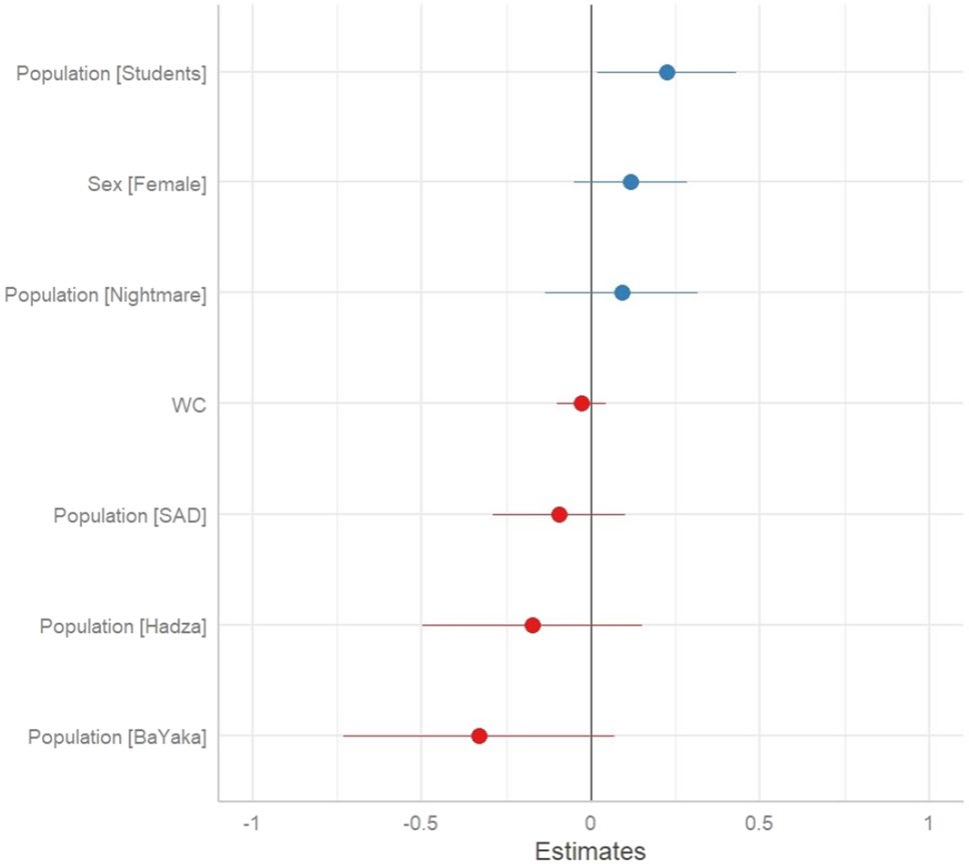
Anxiety dream estimates plot.

After accounting for sex, the word count and participant repeated measures by subject ID, it was found that the student group had more anxiety dream content compared to the reference group. Table 5 and Fig. 4 indicate that no other groups demonstrated a significant difference from the control group. In the following two examples, the dream scenario illustrates the level of anxiety that the subject experiences as he needs to confront challenges alone:

*‘The dream I remember relates to a game that I play. As it only involved myself, there was no one that I knew around, and I remember feeling anxious. I was doing a very difficult mini-game in the game where a bunch of non-player characters were all around me and I needed to hide behind obstacles to stay safe. I remember waking up once I died inside the mini-game’*

**Table 5:** The effect of predictor variables on *anxiety dream content*.

Predictor	Estimate (S.E.)	Confidence interval	P value
Population: BaYaka	− 0.06 (0.03)	(− 0.125, 0.012)	0.10
Population: Hadza	− 0.04 (0.04)	(− 0.116, 0.029)	0.24
Population: Nightmare	0.03 (0.04)	(− 0.040, 0.109)	0.37
Population: SAD	− 0.04 (0.04)	(− 0.117, 0.041)	0.35
Population: Students	0.08 (0.04)	(0.005, 0.163)	0.04*
Sex: Female	0.06 (0.04)	(− 0.017, 0.136)	0.13
Word Count	− 0.02 (0.04)	(− 0.096, 0.056)	0.60

The *Control* is the reference category for *Population*, and *Male* is the reference category for *Sex*. Positive coefficients indicate greater anxiety dream content, while negative coefficients indicate lesser anxiety dream content. The Asterix signifies a significant p value.

Contrary to one of our predictions, no significant differences between the non-clinical group and the Social Anxiety Disorder group were found about the level of anxiety experienced in dreams. However, some dreams illustrate the social isolation these patients are experiencing in their real life, translated by a lack of social support when dangers arise:

*‘I was in an elevator, stuck, alone. I pressed the down button, and then the elevator sped down. I was very scared, I tried to set off the emergency bell. I arrived at the bottom, it was dark and a sheet or blanket fell from the ceiling of the elevator to cover me.’*

In other dreams of this group, people are regarded as hostile, which eventually increases the anxiety level:

*‘I dreamed that I ran into someone I knew at the supermarket. We collided without excusing each other which led to an open conflict. The person in question threatens me, I go to the manager of the store accusing the person of having stolen something (it's not true). Then we walk out of the store and the other begs me to drop my charge of theft. I tell him that I won't go any further and that the newspapers won't know anything because I'm a journalist. The person's mother picks him up. I walk a bit until we go to their place. I explain to the person that I have the feeling of being followed by a man who looks like a shadow, and who watches over me and waits for the moment to seize me. I then understand that this man is death himself!’*

*‘In my dream, I was at my high school. I went into the classroom by myself and two friends (female, 18) that I thought were close to me started isolating me during group work. I worked by myself the entire class while they acted aggressively towards me, at least verbally. I pulled out my chair to go submit my assignment and it hit a person behind me (male, 18). This person is a friend from my primary school. He shouted at me even though I tried to explain to him what happened was just an accident. I used the washroom, and my phone was water-damaged by one of the two girls (may or may not be an accident). I asked her to pay me back, but subconsciously I did not want the refund but instead to have an excuse to hold a conversation with her. It was an unpleasant dream because I thought I was close to them.’*

## Discussion

In the present study, we tested the hypothesis that dreams serve an emotional function that is potentially adaptive by examining dream content from Hadza and BaYaka foragers, who belong to communities characterized by high levels of interpersonal support coupled with greater early-to-midlife mortality (due to predation, resource stress, food and water insecurity, and disease) in comparison to populations in the Global North. We found partial support to the first prediction, that forager dreams exhibit greater community-oriented dream content. Of all the populations examined, only BaYaka reported dreams with significantly more frequent content related to community-orientation and social support amongst family and friends (Table 2 and Fig. 1).

The second prediction, that foragers’ dreams contain more threat related content was supported. Both the BaYaka and Hadza samples demonstrated a greater frequency of mortality and conflict associated dream content compared to the reference group, whereas the other groups did not show such difference (Table 3 and Fig. 2). The prediction that dreams may augment the processing of high threat levels, yet also be characterized by low levels of both anxiety and negative emotions—was supported. The BaYaka exhibited levels of negative emotions in dreams that did not differ from the reference group, while the Hadza exhibited significantly less dream content with negative emotions compared to the reference. As expected, the Nightmare Disorder group also exhibited significantly greater levels of negative emotions in dreams (Table 4 and Fig. 3). A similar pattern was found with anxiety dream content, where the student group during the COVID-19 pandemic was characterized by significantly greater anxiety dream content compared to the reference group, while the BaYaka and Hadza did not differ compared to the reference group (Table 5 and Fig. 4).

### Evidence for an emotional function of dreams in small-scale forager populations

BaYaka and Hadza foragers face several specific hazards. BaYaka communities reside in a rainforest ecology in the Congo Basin, where routine hazards (i.e., specific sources of danger include: (i) intergroup conflict with Bantu fisher-farmers due to perceived trade and labor related debt, (ii) illnesses (malaria, tuberculosis, intestinal parasites), and (iii) extrinsic risks (i.e., broader factors that can increase a person’s overall risk of harm or negative outcomes) of everyday life, including encounters with dangerous animals like snakes, elephants, crocodiles, and gorillas while hunting, fishing, and foraging as well as other hazardous aspects of the forest such as falling limbs/trees and falls while climbing^70^. The BaYaka infant mortality rate in the study region is unknown, but (as measured elsewhere in the region) can be inferred to be around 20 percent^41^. Adult and juvenile mortality is generally relatively high compared to populations with better access to emergency care and biomedical treatment, though precise estimates are currently unknown^41^. A study of death among the Aka in the Central African Republic found that infections and parasitic diseases were the most common causes of death across ages, causing 22 percent of 669 deaths, and diarrhea causing another 21 percent of deaths^71^.

The Hadza reside in a diverse ecological region characterized by rolling hills, grasslands, and acacia commiphora woodland. Hazards for the Hadza include (i) intergroup conflict with the Datoga pastoralists who co-reside in some areas of the landscape and keep large herds of cattle and goats that drink the scare water in the water holes during the dry season and eat much of the vegetation needed to support wildlife, (ii) illnesses (e.g. tuberculosis, malaria, viral diarrhea) that are faced with little access to biomedical treatment, and (iii) extrinsic risks of everyday life that include falling from trees when collecting honey, snakebites, and encounters with predators when hunting or scavenging meat^48^. One study showed that out of 75 deaths, a third of deaths were attributed to illness, with age, childbirth, poisoning or bewitching and homicide, and falling from trees as other causes of death^72^. With respect to mortality, 21% of infants die in the first year of life and 46% of juvenile children die by age 15^72,73^.

Comparatively, populations of the Global North face other types of threats and share different sociocultural values than individuals from small-scale societies. In contrast to collectivistic cultures, like BaYaka and Hadza, most societies of the Global North are strongly individualistic and competitive^74^. People in these societies have less routine face-to-face contact with and imperative cooperative reliance on broad kin networks. At the same time, this individualism shapes many common threats, which are mostly connected to social life (e.g., ostracism and exclusion, loss of status, shame, failure in an exam, etc.), and which are mostly experienced at an individual rather at a collective level. Although recent austerity plans resulted in the reemergence of unemployment, poverty, homelessness, and food insecurity in European and American countries^75^, economic development, public health infrastructure, and access to biomedical care have been linked to comparatively greater life expectancies in the Global North (e.g. 77 years in the U.S. and 80 years in the E.U.), with a larger proportion of deaths occurring in older age from chronic conditions^76–78^.

The present findings provide evidence that when compared to populations in the Global North, foragers disclose a prevalence of community-orientation in their waking life as well as the socially connected themes in their dreams, which may support emotional health. Specifically, our analysis suggests that even in the context of threat, community-orientation—expressed by strong social networks that rely daily on mutual assistance in the context of strong egalitarian social norms—may also play an important role in providing strategies to overcome threats and ultimately achieve emotion regulation. Importantly, an interpretation of BaYaka and Hadza dreams is that foragers activate both the threat simulation and extinction functions of dreaming, which may result in resolution of these threats within their dreams.

### The dysfunctional nature of nightmares

We claim here, in line with other theoretical concepts^17^, that increased threat in dreams (as compared to dreams from healthy controls) does not seem to be functional without a subsequent emotional resolution. For example, patients with nightmare disorder have dreams characterized by recurrent, intense, and highly threatening content that cause significant distress and impairment in social, occupational, or other areas of functioning^56^. Nightmares are dreams with high threat but insufficient emotional resolution. The dreamer cannot find effective solutions for threats, therefore high fear and anxiety impedes emotion regulation and catharsis. According to the threat simulation theory, individuals possess a threat simulation system by which multiple factors (such as, inherited personality traits, threat input throughout adolescent development, current stress levels and recent threat input) regulate dream phenotypes. These inputs can also be attenuated by strong social support networks and egalitarian norms. Previous work has suggested that threatening content in dreams ultimately serve to strengthen waking threat perception skills and threat avoidance behaviors that help to self-cope with the challenging realities of waking life^6,8,79,80^.

The forager data further supports the idea that overcoming threat by way of adaptive emotional responses (in wake or sleep) is a crucial component of an efficient emotion regulation in the face of stressful events. When the presence of threats in dreams is not associated with subsequent emotional resolution, as in recurrent nightmares, dreams seem to lose their emotional processing function. Our results, along with others^81–83^ suggest that nightmares are dysfunctional dreams with high threat simulation coupled with lack of fear extinction.

### Dreams in situations of social isolation or social anxiety

Contrary to the community-oriented character of the BaYaka population, and similar to the increased negative emotions found in nightmares, the dream reports collected from students during the pandemic era were characterized by high levels of anxiety, and sometimes these manifested with themes of isolation and having to confront challenges alone (as depicted in the dream text examples in the “Results” section). For example, dreamers experienced high anxiety because of the presence of hostile people in the narrative, without finding any positive way to deal with such a threat. Our results suggest that dreams of individuals in situations of social isolation or social anxiety do not seem to achieve a sufficient degree of emotional resolution (see also^26^). Whether there is a causal relationship between such a deficient extinction function of dreaming and the symptomatology of anxiety disorders is not clearly elucidated and should be further tested in the future.

### Limitations

There are several limitations to the current study, particularly in regard to the dream content collection among the BaYaka and Hadza populations. Future dream research in such small-scale societies should emphasize not only generating dream data but also including daily reports of activity or evidence of daytime emotion regulation or performance^18^. Accounts for waking life experiences enable a direct analysis of dreams to experiences encountered during the day, which would then allow to test threat or social simulation hypotheses or to make claims related to these hypotheses in general^60^. Correlational studies, such as the one conducted by Sterpenich and colleagues^18^, or interventional studies (i.e., manipulating dream content and observing its effect in wakefulness^25^) offer a closer approximation of the relationship between wake and dream functions. Importantly, observational dream research, including the present study, cannot claim to provide strong evidence for causality between wakefulness and dreams, nor for the directionality of such relationship regarding emotion regulation functions. Finally, as both a point of originality for this work and in distinction from previous work, this study did not test for the daytime emotional state-response, as emotional resolution was assessed in the dream itself.

Dream reports with greater length are more likely to contain sufficient information to accurately describe a dream^29^. Yet, some dream reports from both of these communities were relatively short in length. This can be attributed to dream recounting having to be translated and transcribed into English. Although we made efforts to recount as much detail as possible, dream descriptions could only be paraphrased summaries of dreams distilled through the translator. In addition, it is difficult to assess whether the participant recounting his/her dream was motivated and/or had sufficient practice formulating accurate long-term memories of the dream. Often, inexperienced dream recounters simply answer the questionnaire as is presented to them, which can attribute to dream report bias^80^. Despite the short dream descriptions and less formalized training in dream recounting, the BaYaka and Hadza communities are characterized by a rich storytelling culture and were typically highly motivated to discuss dreams and their interpretations. We also note that these samples are characterized by a stark lack of sexually related activity in dreams. It may well be that for these groups, the lack of recounting dreams of a sexual nature may reflect a taboo placed on descriptions of sexuality in general.

## Conclusion

Here we provide support for the idea that in non-clinical populations with real and perceived threats, dreams may process high threat levels, yet also be characterized by low anxiety and negative emotions. Our results suggest indirectly that dreams can effectively regulate emotions by linking potential dangers with novel, non-fearful dream contexts and can lead to a reduction in feelings of anxiety and other negative emotions, as a form of emotional release or catharsis. In addition, in at least one such community (the BaYaka), emotional catharsis is often achieved by strong social support. Ultimately, if dreaming prepares human beings to face likely challenges and dangers in waking life, then our results are among the first to show these potential functions under evolutionarily relevant socio-ecological conditions.

## Data Availability

The data that support the findings of this study are publicly available on OSF (https://osf.io/7n6kf/).
